# Relationship Reciprocation Modulates Resource Allocation in Adolescent Social Networks: Developmental Effects

**DOI:** 10.1111/cdev.12396

**Published:** 2015-07-31

**Authors:** Stephanie Burnett Heyes, Yeou‐Rong Jih, Per Block, Chii‐Fen Hiu, Emily A. Holmes, Jennifer Y. F. Lau

**Affiliations:** ^1^ University of Oxford; ^2^ University of Birmingham; ^3^ ETH Zürich; ^4^ MRC Cognition and Brain Sciences Unit; ^5^ Karolinska Institutet; ^6^ King's College London

## Abstract

Adolescence is characterized as a period of social reorientation toward peer relationships, entailing the emergence of sophisticated social abilities. Two studies (Study 1: *N *=* *42, ages 13–17; Study 2: *N *=* *81, ages 13–16) investigated age group differences in the impact of relationship reciprocation within school‐based social networks on an experimental measure of cooperation behavior. Results suggest development between mid‐ and late adolescence in the extent to which reciprocation of social ties predicted resource allocation. With increasing age group, investment decisions increasingly reflected the degree to which peers reciprocated feelings of friendship. This result may reflect social‐cognitive development, which could facilitate the ability to navigate an increasingly complex social world in adolescence and promote positive and enduring relationships into adulthood.

Adolescence, the period between childhood and adulthood, is characterized by changes in social behavior (Spear, 2000; Steinberg, 2008). Across several species, individuals in adolescence undergo a process of “social reorientation” entailing increased affiliation with peers, relative to family members (Lorme, Bell, & Sisk, 2013; Nelson, Leibenluft, McClure, & Pine, 2005).

During human adolescence, important changes take place within peer relationships. Increasing emphasis is placed on friendship intimacy and reciprocity (Youniss, 1982), and there is a rise in the importance of loyalty, commitment, and trust (Bigelow & La Gaipa, 1980). These changes in expectations of friendship are thought to impact the way in which adolescents manage and define their peer relationships. For example, an observed decrease in the number of self‐reported close friends during adolescence is hypothesized to result from increased selectivity due to greater demands for intimacy and reciprocity (Urberg, Değirmencioğlu, Tolson, & Halliday‐Scher, 1995). Taken together, this body of work suggests that during adolescence, changing perceptions and awareness of friendship qualities such as reciprocity may influence the way in which adolescents interact within their peer relationships.

It is proposed that the changes described above, which are observed in the context of adolescent peer relationships, are contingent upon developmental advances in social perspective taking (Selman, 1980). In one study (Selman, 1980, pp. 43–46) open‐ended interview responses to social dilemmas were elicited from participants aged 9–17 years. Responses were coded with reference to a metric of the highest level of perspective taking used with competence, where Level 2 indicates simple reciprocal perspective taking (ability to grasp self *or* other's perspective), Level 3 indicates mutual perspective taking (ability to simultaneously grasp self *and* other's perspective), and Level 4 is indicative of a conceptual, multilevel understanding of interpersonal relations. From childhood to early adolescence (from ages 9–10 to 12–14 years), there was an increase in the proportion of Level 3 as compared to Level 2 responses, and transitioning into late adolescence (age 16–17 years), responses were predominantly at Levels 3 and 4. Thus, the study provided initial evidence that social perspective taking continue to develop during adolescence (see also Gurucharri & Selman, 1982).

Converging evidence from experimental paradigms suggests protracted development during adolescence in social‐cognition (Burnett & Blakemore, 2009). In one study, child, adolescent, and adult participants (aged 7–27 years) played a computerized perspective‐taking game in which their task was to move objects between shelves as instructed by a computer avatar. Results showed an increase throughout adolescence and into early adulthood in the tendency to automatically take into account the perspective or communicative intention of the computer avatar (Dumontheil, Apperly, & Blakemore, 2010). Other experimental studies have utilized game theory paradigms, in which principles such as fairness and altruism are operationalized via conversion into a common monetary currency that participants share, cooperate, or compete for during structured interactions (Rilling & Sanfey, 2011). Using these paradigms, studies have shown evidence for development during late childhood and adolescence in the tendency to take into account the perspectives, motives, and relative contributions of self and other (Almås, Cappelen, Sørensen, & Tungodden, 2010; Güroğlu, van den Bos, & Crone, 2009; Van den Bos, Westenberg, van Dijk, & Crone, 2010). For example, one study used a modified Ultimatum Game to show that investment decisions were increasingly modulated by the perceived ability of an unknown peer to reject selfish offers between ages 9 and 18 years (Güroğlu et al., 2009, Experiment 2). Results from these experimental studies align with the observational and structured interview findings summarized above suggesting advances in certain social‐cognitive abilities up to and throughout the period of adolescence. However, while prior experimental studies provide important insights, they primarily rely upon interactions with unknown peers or computer stooges. Experimental research is needed that systematically investigates laboratory‐based measures of social behavior (e.g., from game theory) in the context of real‐world adolescent peer relationships.

A more nuanced empirical picture may emerge when social behavior is investigated in the context of authentic, established relationships. In authentic relationships, interactions unfold across extended periods of time, and individuals derive long‐term benefits of sharing and cooperating when they can expect future direct or indirect reciprocation from others (Frank, 1998; Gardner & Foster, 2008; Hammerstein & Leimar, 2006; Nowak & Sigmund, 1998). Furthermore, during child and adolescent development, peer relationships provide a platform to practice and refine advanced social skills of cooperation, competition, and compromise (Youniss, 1982). Therefore, investigating social behavior in the context of authentic peer relationships may enable not only more ecologically valid assessments of social cognitive abilities, but may also show greater sensitivity to subtle changes within a developmental period. To accomplish this empirical goal, paradigms are needed that are capable of quantifying relevant features of real‐world peer relationships, and such paradigms should be tied to experimental measures of social behavior.

A recently developed paradigm combines quantitative social network analysis techniques for mapping real‐world relationships (Dekker, Krackhardt, & Snijders, 2007) with an experimental measure of prosocial cooperation behavior (Harrison, Sciberras, & James, 2011), where prosocial behavior is defined as voluntary behavior that benefits others (Eisenberg, Fabes, & Spinrad, 2007). In this recent study, adult members of a real‐world social network (a research laboratory) completed an anonymous Social Network Questionnaire (SNQ) that comprised questions assessing feelings of closeness and companionship toward network peers. Responses were summed to yield a continuous measure of the perceived strength of social ties linking pairs of individuals (dyads) within the network, as well as the degree to which perceived social tie strength was reciprocated (Harrison et al., 2011). Subsequently, participants had the opportunity to earn money for themselves and a subset of their network peers in a physical effort‐based task. Results showed that the strength of perceived social ties linking each participant to their network peers predicted the amount of money participants were willing to earn for their peers, at physical cost to themselves. Furthermore, the extent to which social ties were reciprocated predicted additional variance in investment. That is, participants' resource allocation decisions were sensitive to the degree to which feelings of closeness were reciprocated by their peers.

In the current study, we adapted this paradigm to investigate development during adolescence in the extent to which reciprocation of social ties within school‐based social networks modulated prosocial resource allocation. Participants completed an SNQ, modified from Harrison et al. (2011), to take into account features of adolescent school‐based relationships. To increase feasibility in a school setting, our measure of resource allocation was a modified one‐shot Dictator Game. In this simple game theory paradigm, participants decide how much of an endowment of goods (e.g., money) to give to others, at cost to themselves. Thus, the task measures the extent to which participants are willing to share resources. Previous research has shown that Dictator Game resource allocation decreases with increasing social distance from peers, suggesting that this measure is sensitive to the strength of social ties in real‐world social networks (Goeree, McConnell, Mitchell, Tromp, & Yariv, 2010).

We conducted two studies. In Study 1 (*N *=* *42), we mapped two social networks: midadolescent (*M*_age_ = 14) and late adolescent (*M*_age_ = 17). In Study 2 (*N *=* *81), we mapped four social networks: midadolescent (*M*_age_ = 14), later midadolescent (*M*_age_ = 15), and late adolescent (two networks; *M*_age_ = 16). In Study 1, each modified Dictator Game (mDG) point was worth real money, so participants had to decide how much of an opportunity cost they were willing to bear in order to share points. In Study 2, money was fictitious. Across both studies, we predicted that relationship strength would modulate resource allocation across age groups; that is, participants would invest more in recipients to whom they reported stronger social ties (Goeree et al., 2010; Harrison et al., 2011). Based on the evidence reviewed above (Dumontheil et al., 2010; Selman, 1980; Youniss, 1982), in Study 1 we predicted that the reciprocation of social ties would modulate resource allocation differentially across age groups; that is, in late adolescence, but not in midadolescence, individuals would invest more in individuals who reported stronger *reciprocal* social ties. In Study 2, we predicted a replication of Study 1 findings; in addition, the inclusion of three developmental time points enabled us to track hypothesized continuous (e.g., linear) change within the period of adolescence in the behavioral impact of relationship reciprocation. That is, we predicted that the extent to which reciprocation of social ties on the SNQ would modulate Dictator Game resource allocation would increase with increasing age group. Finally, both studies explored the impact of additional variables (Study 1 and Study 2: Machiavellian orientation [Christie & Geis, 1970], duration of acquaintance; Study 2: affluence [Currie et al., 2004], self‐reported empathy [Davis, 1980], and number of best friends present in the current network) that could theoretically modulate prosocial behavior in the current study (Edele, Dziobek, & Keller, 2013; Goeree et al., 2010; Nettle, Colléony, & Cockerill, 2011; Spitzer, Fischbacher, Herrnberger, Grön, & Fehr, 2007) and that could, if they differed significantly between groups, give rise to confounding differences in comparisons across age groups.

## Study 1

### Method

#### Participants

We included 42 participants from a coeducational private school in a village in South East England based on cohort year group (grade level): Year 9 (midadolescent network): *n *=* *23 (11 female), *M* (*SD*) age = 14.46 (.29) years, range = 13.88–14.79; Year 12 (late adolescent network): *n *=* *19 (9 female), *M* (*SD*) age = 17.22 (.26) years, range = 16.91–17.97 (see Table 1). Participants were selected in consultation with a teacher such that individuals within each network spent class time together each week as a group. We obtained parent or guardian consent for all participants. Data were collected during May 2012. The study was approved by the University of Oxford research ethics committee and was carried out in accordance with the provisions of the World Medical Association Declaration of Helsinki.

**Table 1:** Study 1 Descriptives for Each Year Group Network

	Year 9 Midadolescence			Year 12 Late adolescence			Group comparison
	*M* (*SD*)	Range	*N*	*M* (*SD*)	Range	*N*	*t*(*df*), *p*
Age (years)	14.46 (0.26)	13.88 to 14.79	23	17.22 (0.29)	16.91 to 17.97	19	
Kiddie Mach	51.74 (7.10)	41 to 70	23	55.47 (10.4)	42 to 85	19	*t*(40) = 1.38, *p *=* *.176
mDG: Points to self	13.88 (27.50)	0 to 100	23	28.36 (35.30)	0 to 100	19	*t*(34)^a^ = 1.46, *p *=* *.154

	Density *M* (SEM)	Range	*N*	Density *M* (SEM)	Range	*N*	*t*(*df*), *p*
mDG: Points to others	3.91 (.10)	0 to 15	506	3.98 (.21)	0 to 22.22	342	*t*(846) = .28, *p *=* *.388
SNQ relationship strength^b^	0.41 (.01)	−.13 to 1	506	0.33 (.01)	−.25 to 1	342	*t*(846) = 4.36, *p *<* *.001
SNQ relationship reciprocation^c^	0.15 (.01)	−.88 to .88	506	0.15 (.01)	‐.63 to .63	342	*t*(846) = 1.59, *p *=* *.056
Duration of acquaintance (years)^d^	3.04 (.12)	1 to 15	506	2.6 (.11)	.67 to 17.32	342	*t*(846) = 3.01, *p *=* *.001

Note

The upper half of the table shows nonnetwork descriptives while the lower half of the table shows descriptives for network data. Network data were compared across groups using *t* tests based on bootstrapped mean density and standard error, with degrees of freedom based on *N* observations across groups. mDG = modified Dictator Game; SNQ = Social Network Questionnaire.

^a^Degrees of freedom reduced to take into account inequality of variances. ^b^Relationship strength values represent perceived strength of unidirectional social ties. Values close to one reflect strong relationships; negative values or values close to zero reflect negative or neutral perceived relationships, respectively. ^c^Reciprocation values represent the extent to which relationships are positively reciprocated. Positive values reflect greater in‐ than out‐link strength. ^d^For Year 9, maximum duration of acquaintance exceeds maximum age. Most likely, a participant rounded up to the nearest year when reporting duration of acquaintance.

#### Measures

##### Modified Dictator Game

Participants played an mDG to measure how much money (in the form of shopping vouchers) they were willing to give to other network members, at cost to themselves. The task is structurally equivalent to a one‐shot Dictator Game (see Goeree et al., 2010) with multiple recipients. We provided participants with a table in which all members of the network were listed. Each participant had 100 points (1 point = £0.01) to distribute across the list. Instructions stated that there was no minimum point limit for each individual. Therefore, participants could choose to keep all the money by allotting 100 points to themselves, or to share points with network peers. We informed participants that the total amount of money they would receive would be determined by summing the points they allotted themselves and points other network members gave to them.

##### Social Network Questionnaire

We adapted a SNQ (Harrison et al., 2011) for adolescent participants. Participants rated network members on eight SNQ items. Five items provided binary data (yes = 1, no = 0): (a) whether the participant had a familial tie with each network member, (b) whether the participant had a current romantic relationship with each network member, (c) whether the participant spent time outside of school with each network member, and (d) whether the participant had ever chosen to academically work with each network member; (e) whether the participant would choose to confide personal bad news in each network member, and Three items provided ordinal or continuous data: (f) nature of relationship with each network member (4 = *best friend*, 3 = *good friend*, 2 = *friend*, 1 = *acquaintance*, 0 = *no relationship*, −1 = *negative relationship*), (g) how much the participant trusted each network member, relative to a random person from another school (1 = *more than*, 0 = *the same as*, −1 = *less than*), and (h) duration of acquaintance with each network member (years). The five relationship strength items (c, d, e, f, g) assessed various constructs (e.g., companionship, intimacy) that contribute to relationship strength (Bagwell & Schmidt, 2013). Additional items (a, b, h) were included to measure potential confounding effects of direct familial ties, romantic relationships, and duration of acquaintance. Since there were only three distant familial ties and two romantic relationships across the sample, these two variables were dropped from subsequent analyses. Participants also provided information regarding their gender.

##### Kiddie Mach

The Kiddie Mach (Nachamie, 1970) is a 20‐item self‐report questionnaire for children and adolescents derived from the adult Machiavellianism Scale–IV (Christie & Geis, 1970), which assesses lack of faith in human nature, manipulation, dishonesty, and distrust. The measure was included to investigate potential effects of Machiavellian orientation on mDG investment. Items include: “Most people are good and kind” and “Sometimes you have to hurt other people to get what you want.” Agreement with Machiavellian items is indicated on a scale from 1 (*strongly disagree*) to 5 (*strongly agree*). Non‐Machiavellian items are reverse scored such that high scores indicate disagreement and therefore Machiavellianism. The measure shows good internal reliability in adolescent samples (Sutton & Keogh, 2001). Previously, greater scores on this measure have been associated with reduced prosocial resource allocation on an mDG (Spitzer et al., 2007).

#### Experimental Procedure

Participants in each group completed the measures using pen and paper in a large testing room that afforded privacy for each individual. The measures took approximately 30 min to complete. After testing, all participants received a £5 shopping voucher for their participation in the mDG and a separate task unrelated to the present research.

#### Statistical Procedures

Below, we describe data processing procedures, followed by statistical analyses.

##### Data Processing

###### Modified Dictator Game

Investment in the mDG was used to create the following variables. (a) *Points allocated to self*. For each year group, we calculated the number of points allocated to self. (b) *Points allocated across the network*. For each year group, we created matrices in which the amount donated by a given participant to their classmates was listed in rows and the amount received by a given participant from their classmates was listed in columns.

###### Social Network Questionnaire

Responses on the SNQ were used to create the following variable matrices. (a) *Relationship strength*. For each year group, a relationship strength matrix representing the strength of social ties reported by a given participant toward other members of their network was created by summing responses to all five SNQ relationship strength items and, for ease of interpretation, dividing by eight the highest possible summed score. Thus, relationship strength matrix values (i.e., link weights between −.25 and 1) represent the strength of each social relationship: Negative values or values closer to 0 reflect more negative or neutral relationships, while values closer to 1 reflect stronger relationships. Matrix rows represent out‐links, or how strong a relationship a given participant reports toward others. Matrix columns represent in‐links, or how strong a relationship others report toward the participant. (b) *Reciprocation*. For each year group, a reciprocation matrix encapsulating the extent to which individuals reciprocated social ties was created by subtracting the relationship strength matrix for each year group from the respective transposed relationship strength matrix. As such, the reciprocation matrix is symmetrical with inverted sign; that is, for every positive entry there is a negative entry with the same absolute value on the other side of the diagonal of the matrix, and the sum of all elements of the matrix is necessarily zero. Reciprocation matrices make explicit the extent to which dyadic relationship strength is reciprocated: More negative values reflect relationships for which out‐link exceeds in‐link strength, while more positive values reflect relationships for which in‐link exceeds out‐link strength. (c) *Gender giving and gender receiving*. For each year group we created “gender giving” and “gender receiving” matrices by entering individual gender values (0 = female, 1 = male) across matrix rows and columns respectively. (d) *Duration of acquaintance*. For each year group we created a duration of acquaintance matrix using the SNQ duration of acquaintance item.

###### Kiddie Mach

Total Mach scores were calculated for each participant. In addition, for each year group we created “Mach giving” and “Mach receiving” matrices by entering individual Mach scores across matrix rows and columns, respectively.

##### Statistical Analyses

The data analysis strategy was as follows. First, *t* tests were conducted to investigate descriptive differences across networks. Second, multiple regressions were conducted to evaluate the impact of network and individual difference variables on mDG investment separately in each year group. Third, multigroup multiple regression was conducted to evaluate the impact of network and individual difference variables identified as of potential interest in Steps 1 and 2 on investment across the entire sample, and to pinpoint whether this impact varied as a function of year group, as a proxy for developmental age.

###### Descriptive differences across networks

*T* tests were conducted comparing the following variables across groups: mDG points allocated to self, mDG points allocated across the network, SNQ relationship strength, SNQ reciprocation, duration of acquaintance, and total Mach score. For comparison of network data (i.e., mDG points allocated across the network, SNQ relationship strength, SNQ reciprocation, and duration of acquaintance) a bootstrapping approach was used to compute standard errors based on mean network density, and subsequently, *t* tests were conducted comparing mean network density between groups (Snijders & Borgatti, 1999). Note that for network data, degrees of freedom are equal to the number of tie variables across networks. Statistical significance was *p *<* *.05 two‐tailed.

###### Multiple regression predicting resource allocation within networks

Multiple regression was conducted to evaluate the relationship between social network and individual difference variables and mDG point allocation in each year group. This entailed the use of statistical procedures capable of accounting for statistical dependence among observations, since didactic interaction data in social networks are based upon nonindependent observations: Measuring participant X's relationship with all individuals in the network requires repeat observations of X. Thus, observations of X are nonindependent. We utilized a statistical method capable of addressing nonindependent data: multiple regression with quadratic assignment procedure (MRQAP; Dekker et al., 2007; Hemelrijk, 1990; Krackhardt, 1988) executed in UCINET (version 6.415; Borgatti, Everett, & Freeman, 2002). MRQAP implements linear regression for matrix data. Regression coefficients can be interpreted as in ordinary least squares regression, although standard errors are calculated differently. Briefly, MRQAP regresses a dependent variable matrix upon explanatory variable matrices to obtain regression coefficients. Subsequently, row by column positions of the residuals resulting from the regression are randomly permuted (i.e., double Dekker semipartialing) to obtain regression coefficients of the permutations, which form the sampling distribution under the null hypothesis. The original regression coefficients are compared to this null distribution for hypothesis testing. We utilized UCINET's MRQAP with double Dekker semipartialing and 10,000 permutations to regress Year 9 and Year 12 mDG point allocation matrices against their respective year group explanatory variables: SNQ relationship strength, SNQ reciprocation, gender giving, gender receiving, duration of acquaintance, Mach giving, and Mach receiving. Statistical significance was *p *<* *.05, two‐tailed for nondirectional and one‐tailed for directional hypotheses. Directional hypotheses related to effects on point allocation of SNQ social tie strength (Harrison et al., 2011), gender (Balliet, Li, Macfarlan, & Van Vugt, 2011), Mach (Spitzer et al., 2007), and SNQ reciprocation (Dumontheil et al., 2010; Güroğlu et al., 2009; Selman, 1980). We dropped nonsignificant variables in a stepwise manner, starting with the variable for which the two‐tailed *p* value was closest to 1.

###### Multiple regression predicting resource allocation across networks

Multigroup multiple regression (i.e., MG‐MRQAP) was conducted to test directly for interactions between year group and the relation between mDG point allocation and explanatory variables described above. This first entailed the creation of full‐sample matrices for each of the dependent and explanatory variables described above. These data have a multilevel structure, with participants (*N *=* *42) nested in classes (Year 9, Year 12). We estimated regression parameters of interest using linear regression with pooled data from both classes and calculated standard errors by permuting each of the two matrices. Note that for the estimation of multiple classes simultaneously, a different, more efficient permutation method was chosen that tends to overestimate the size of the standard errors. To account for nesting of participants in classes, the procedure allowed permutation of rows and columns within classes only; in addition, the procedure allowed intercepts for the different classes to vary independently (i.e., a fixed effects approach by class). Thus, we could examine a network level variable (year group) for interactions with explanatory variables (e.g., reciprocation). Multiplying all explanatory matrix values by the appropriate network level variables enabled us to test whether the effect size of an explanatory variable depended on a network level variable. We coded the network level variable “year group” 0 and 1 for participants in Years 9 and 12, respectively, and ran the analysis in R (R Development Core Team, 2011). As for single‐group MRQAP, statistical significance was *p *<* *.05, two‐tailed for nondirectional and one‐tailed for directional hypotheses. Directional hypotheses related to effects on point allocation of SNQ relationship strength, gender, Mach, and the interaction between age group and SNQ reciprocation. We dropped nonsignificant variables in a stepwise manner, starting with the variable for which the two‐tailed *p* value was closest to 1. An exception to this rule was variables specifying main effects for which the interaction term was still in the model. In this case, exclusion of the main effect took place after exclusion of the interaction term. Predictors entered in the initial regression model were (a) variables that differed significantly between groups based on *t*‐test results comparing descriptives across groups, (b) variables that had an impact on investment in either group based on single‐group MRQAP results, and (c) terms included to test for interactions between all predictor variables and group (for a full list of predictor variables, see content and footnote of Table 3).

### Results

#### Descriptive Differences Across Networks

Groups did not differ on points allocated to self, *t*(34) = 1.46, *p *=* *.154; points allocated to others, *t*(846) = .29, *p *=* *.388; SNQ reciprocation, *t*(846) = 1.59, *p *=* *.056; or total Mach score, *t*(40) = 1.38, *p *=* *.176 (see Table 1). There were group differences in duration of acquaintance, *t*(846) = 3.01, *p *=* *.001, and SNQ relationship strength, *t*(846) = 4.36, *p *<* *.001, such that Year 9 participants had been mutually acquainted for longer than Year 12 participants and reported greater relationship strength on average (see Table 1; Figures S1 and S2 in the online Supporting Information depict Year 9 and Year 12 networks based on relationship strength).

#### Multiple Regression Predicting Resource Allocation Within Networks

##### Year 9 model

The final Year 9 point allocation MRQAP model (*R* ^2^_adj_ = .36, *p *<* *.001) included SNQ relationship strength (*b *=* *4.84, *p *<* *.001), gender giving (*b *= −.80, *p *=* *.037), and gender receiving (*b *=* *.39, *p *=* *.006; see Table 2). Ceteris paribus, participants in Year 9 gave more points to those with whom they reported stronger social ties, female participants gave more points to others in their network than did male participants, and male participants received more points than did female participants.

**Table 2:** Study 1 Multiple Regression Models Predicting Resource Allocation Within Networks

Independent variable	Unstandardized regression coefficient	Standardized regression coefficient	*p* value
Year 9: Midadolescence MRQAP final model: *R* ^2^_adj_ = .36, *p *<* *.001			
Intercept	2.17	.00	< .001
Relationship strength	4.84	.56	< .001^a^
Gender giving	−0.80	−.18	.037^a^
Gender receiving	0.39	.08	.006
Year 12: Late adolescence MRQAP final model: *R* ^2^_adj_ = .24, *p *<* *.001			
Intercept	6.76	.00	< .001
Relationship strength	6.06	.40	< .001^a^
Reciprocation	2.09	.12	.032^a^
Gender giving	−1.47	−.19	.027^a^
Mach giving	−0.07	−.19	.032^a^

Note

Multiple regression with quadratic assignment procedure (MRQAP) was used to predict modified Dictator Game point allocation based on individual difference and network variables. Independent variables eliminated from the Year 9 model due to nonsignificance were Mach receiving (*b *= −.00, *p *=* *.499), reciprocation (*b *= −.09, *p *=* *.432), Mach giving (*b *=* *.01, *p *=* *.402), and duration of acquaintance (*b *= −.03, *p *=* *.209). Independent variables eliminated from the Year 12 model due to nonsignificance were gender receiving (*b *=* *.27, *p *=* *.251), Mach receiving (*b *=* *.03, *p *=* *.111), and duration of acquaintance (*b *= −.15, *p *=* *.088).

^a^Denotes a one‐tailed *p* value; all other *p* values are two‐tailed.

##### Year 12 model

The final Year 12 point allocation model (*R* ^2^_adj_ = .24, *p *<* *.001) included SNQ relationship strength (*b *=* *6.06, *p *<* *.001), reciprocation (*b *=* *2.09, *p *=* *.032), gender giving (*b *=* *−1.47, *p *=* *.027), and Kiddie Mach giving (*b *= −.07, *p *=* *.032; see Table 2). Ceteris paribus, participants in Year 12 gave more points to those toward whom they reported stronger social ties and to individuals who positively reciprocated social ties (i.e., those who reported a stronger relationship with the participant than the participant reported toward them). Female participants gave more points to others in their network than did male participants, and individuals who scored higher on Mach gave fewer points to others in their network than did those who scored lower on this measure.

#### Multiple Regression Predicting Resource Allocation Across Networks

We regressed mDG point allocation across the entire sample (*N *=* *42) on explanatory variable matrices using MG‐MRQAP. In the final point allocation model (*R* ^2^_adj_ = .26, *p *<* *.001), significant predictor variables were SNQ relationship strength (*b *=* *4.82, *p *<* *.001), SNQ Reciprocation × Year Group (*b *=* *2.64, *p *=* *.040), and gender giving (*b *=* *−1.18, *p *=* *.003; Table 3). Across the entire sample, ceteris paribus, participants gave more to those toward whom they reported stronger social ties, male participants gave fewer points to others in their network than did female participants, and, relative to Year 9 participants, Year 12 participants gave more to individuals who positively reciprocated social ties (see Table 3).

**Table 3:** Study 1 Multiple Regression Models Predicting Resource Allocation Across Networks

Independent variable	Unstandardized regression coefficient	*p* value
Study 1 MG‐MRQAP model: *R* ^2^_adj_ = .255, *p *<* *.001		
Year 9 intercept	2.58	< .001
Year 12 intercept (reference Year 9)	−0.23	.552
Relationship strength	4.82	< .001^a^
Reciprocation	−0.17	.820
Reciprocation × Year group	2.64	.040^a^
Gender giving	−1.18	.003^a^

Note

Multigroup multiple regression with quadratic assignment procedure (MG‐MRQAP) was implemented to predict modified Dictator Game point allocation based on individual difference and network variables and terms specifying their respective interactions with year group (Year 9 and Year 12). Independent variables eliminated from the model were Gender Receiving × Year Group (*b *= −.02, *p *=* *.493), Gender Giving × Year Group (*b *= −.41, *p *=* *.354), Duration of Acquaintance × Year Group (*b = *−1.17, *p *= .134), duration of acquaintance (*b *= −.08, *p *=* *.078), gender receiving (*b *=* *.29, *p *=* *.909), Mach Giving × Group (*b *= −.09, *p *=* *.071), Mach giving (*b *= −.04, *p *=* *.105), and Relationship Strength × Year Group (*b = *1.83, *p *=* *.062). Note that for the multigroup models in the current study, it is not appropriate to report standardized regression coefficients. In order to investigate interactions, the Year 12 intercept and reciprocation terms were retained. That is, the procedure for eliminating variables was to exclude the variable with the largest *p* value, unless this belonged to a main effect for which the interaction term was still in the model. In this case, exclusion of main effects took place after exclusion of interaction terms.

^a^Denotes a one‐tailed *p* value; all other *p* values are two‐tailed.

### Discussion

Results indicate that in both midadolescent (*M*_age_ = 14) and late adolescent (*M*_age_ = 17) networks, individuals invested more in peers toward whom they reported stronger social ties. This is consistent with a previous study in adults, in which cooperative investment in a physical effort‐based task increased as a function of dyadic social tie strength (Harrison et al., 2011). The current finding is also consistent with a previous study in adolescents, which showed that cooperative investment decreased with increasing social distance (Goeree et al., 2010). Additionally, in the current study, we found that, relative to the midadolescent network, individuals in the late adolescent network invested more in peers who *reciprocated* strong ties. That is, late adolescents, but not midadolescents, were behaviorally sensitive to the degree to which others reciprocated their feelings of friendship, and adjusted their resource allocation strategy accordingly.

An intuitive way to interpret these results is as follows. In the older, late adolescent (Year 12) network, giving is maximal when I like you a lot *and* when you like me a lot (e.g., social tie strength = 1 in both directions). Giving is least when I dislike you a lot *and* you dislike me a lot (e.g., social tie strength = −0.25 in both directions). Between these extremes there are gradients—the less I like somebody, the less I give to them, but also, the less I am liked by them, the less I give to them. In contrast, in the younger, midadolescent (Year 9) network, giving is simply a function of how much I like you, and does not vary as a function of how much *you* like *me*.

This result is consistent with observations that the understanding of interpersonal reciprocity and mutuality continues to develop during adolescence (Selman, 1980), and with experimental evidence that social cognitive abilities, including perspective taking, continue to develop during adolescence (Dumontheil et al., 2010; Güroğlu et al., 2009). The current data constitute interesting preliminary evidence for differences between mid‐ and late adolescent networks in behavioral sensitivity to relationship reciprocation in the context of authentic, face‐to‐face peer relationships. However, to dissociate hypothesized continuous (e.g., linear) developmental advances in the behavioral impact of reciprocity from the effects of baseline, uncontrolled differences in individual characteristics between networks, comparison of more than two developmental time points is needed. Therefore, Study 2 examined developmental reciprocity in greater detail by directly comparing three cross‐sectional time points within a larger mid‐ to late adolescent sample. We predicted that the degree to which participants reciprocated social ties would modulate resource allocation to a greater extent in late adolescent, relative to midadolescent, networks.

In Study 1, two descriptive differences were observed across groups: First, duration of acquaintance was higher in midadolescence than in late adolescence. However, since this variable was eliminated due to nonsignificance from the multigroup regression model, it cannot account for group differences in patterns of investment. Second, SNQ relationship strength was greater in midadolescence than in late adolescence. However, since the interaction between age group and relationship strength was nonsignificant in the final multigroup regression model, the group difference in this variable cannot account for the pattern of findings. In Study 2, we explored the generalizability of this finding.

In Study 1, individual difference variables exerted subtle effects on investment patterns. Based on single‐ and multigroup regression models, females tended to give more points than males, consistent with evidence that females tend to be more cooperative than males in mixed‐gender contexts (Balliet et al., 2011). Machiavellianism had a weak, negative impact on investment decisions in the late adolescent network, aligning with evidence that individuals scoring more highly on this measure value personal gain over benefits for others (Christie & Geis, 1970; Wilson, Near, & Miller, 1996) and with a previous study in adults using an mDG (Spitzer et al., 2007). However, the absence of an effect in the midadolescent network suggests that the current finding should be interpreted with caution. Furthermore, in addition to Machiavellianism, there are other individual characteristics that may influence investment decisions, such as wealth (Nettle et al., 2011), empathy (Edele et al., 2013), and the number of close friends present in the current network (Goeree et al., 2010), and these could also contribute to the observed differences in investment patterns between networks. Therefore, in Study 2 we included three additional measures: the Interpersonal Reactivity Index (IRI; Davis, 1980), the Family Affluence Scale (Currie et al., 2004), and a peer nominations questionnaire to investigate potential group differences in self‐reported empathy, individual levels of wealth, and the number of best friends present in the current network, respectively, while a single‐gender sample was used to increase statistical power by eliminating gender effects as observed in Study 1.

## Study 2

### Method

#### Participants

We recruited 81 participants from a single‐sex (female) state school in a town in South East England based on cohort year group (grade level): Year 9: *n *=* *13, *M* (*SD*) age = 14.10 (.36) years, range = 13.48–14.47; Year 10: *n *=* *24, *M* (*SD*) age = 15.10 (.38) years, range = 14.53–16.09; Year 11A: *n *=* *25, *M* (*SD*) age = 15.96 (.24) years, range = 15.50–16.38; Year 11B: *n *=* *19, *M* (*SD*) age = 16.00 (.28) years, range = 15.58–16.41 (i.e., Year 11A and Year 11B networks are different classes from the same grade level). Participants were selected in consultation with a teacher such that individuals within each network spent class time together each week as a group. Data were collected during February 2013. We obtained parent or guardian consent for all participants. The study was approved by the University of Oxford research ethics committee and was carried out in accordance with the provisions of the World Medical Association Declaration of Helsinki.

#### Measures

##### Modified Dictator Game

Participants played an mDG to measure how much money they would be willing to give to other network members, at cost to themselves. The mDG was identical to that in Study 1 except we instructed participants to imagine they had £100 to distribute among their network. The use of hypothetical rather than real money was motivated by the preferences of the school from which the sample was recruited and based on evidence that hypothetical money effectively motivates participant behavior (Miyapuram, Tobler, Gregorios‐Pippas, & Schultz, 2012).

##### Social Network Questionnaire

The SNQ was identical to that in Study 1, with the following three exceptions: (a) The duration of acquaintance item was altered for speed and ease of completion. Participants rated whether they had known each network member since primary school (= 1) or secondary school (= 0). This variable was labeled “Duration 1.” Participants also reported for how long (in years) they had attended the current school. This variable was labeled “Duration 2.” (b) The romantic ties item was dropped due to low positive response rates in Study 1. (c) An item was included to assess conflict resolution. Along with intimacy and companionship, conflict resolution is an important dimension of friendship and contributor to relationship strength (Bagwell & Schmidt, 2013). For each network member, participants rated how much effort they would expend to resolve a disagreement (1 = *great effort*, 0 = *small effort*, −1 = *would not care*). There were no familial ties across the sample, and therefore the familial ties variable was dropped from analysis.

##### Interpersonal Reactivity Index

The IRI (Davis, 1980) was administered to investigate the relationship between prosocial investment and self‐reported empathy. The IRI is a 28‐item multidimensional self‐report measure of dispositional empathy with four 7‐item subscales: perspective taking (PT; tendency to spontaneously adopt the psychological point of view of others in everyday life; e.g., “I sometimes try to understand my friends better by imagining how things look from their perspective”), empathic concern (EC; tendency to experience feelings of sympathy and compassion for unfortunate others; e.g., “I often have tender, concerned feelings for people less fortunate than me”), personal distress (PD; tendency to experience distress and discomfort in response to extreme distress in others; e.g., “Being in a tense emotional situation scares me”), and fantasy (F; tendency to imaginatively transpose oneself into fictional situations; e.g., “When I am reading an interesting story or novel, I imagine how I would feel if the events in the story were happening to me”). Statements are scored on a 5‐point scale from A (*does not describe me very well*) to E (*describes me very well*). Good internal reliability and test–retest stability for each subscale have been reported for this measure in adults (Davis, 1980). Developmental research has shown a similar factor structure for this measure in adolescent participants, with age‐associated increases in test–retest stability (Davis & Franzoi, 1991). Previous research has shown a relation between IRI EC and prosocial resource allocation (Edele et al., 2013). In the current study, we focused our analysis on the EC and PT subscales.

##### Family Affluence Scale II (FASII)

The FASII (Currie et al., 2004) is a four‐item measure of socioeconomic status suitable for completion by adolescents. This measure was administered to assess potential effects on investment of differing levels of wealth. Items include: “Does your family own a car or van?” (responses: No; Yes, one; Yes, two or more) and “Do you have your own bedroom?” (responses: No; Yes). Reliability and validity has been reported in detail elsewhere; for example, studies have shown high child–parent agreement (Currie et al., 2008). Previous research has shown an impact of differing levels of wealth on Dictator Game investment (Nettle et al., 2011).

##### Kiddie Mach

The Kiddie Mach (Nachamie, 1970) was administered as in Study 1.

##### Best Friends

A peer nominations questionnaire was administered in which participants listed their five best friends (in any setting). This measure was administered to assess potential effects on mDG investment of the number of self‐reported close friendships in the current network.

#### Experimental Procedure

Participants in each group completed the measures using pen and paper in a large testing room that afforded privacy for each individual. The measures took approximately 40 min to complete.

#### Statistical Procedures

Below, we describe data processing procedures, followed by statistical analyses.

##### Data Processing

###### Modified Dictator Game

As in Study 1, we used mDG responses to create two variables: money allocation to self and money allocation to others. The latter is a matrix.

###### Social Network Questionnaire

Responses on the SNQ were used to create the following variables. (a) *Relationship strength*. For each year group, a relationship strength matrix representing the strength of social ties reported by a given participant toward other members of their network was created by summing responses to all six SNQ relationship strength items and, for ease of interpretation, dividing by nine the highest possible summed score. Thus, relationship strength matrix values (i.e., link weights between −.33 and 1) represent the strength of each social relationship. (b) *Reciprocation*. For each year group, a reciprocation matrix was created by subtracting the relationship strength matrix (out‐link values) for each year group from a transposed relationship strength matrix (in‐link values), as in Study 1. (c) *Duration of acquaintance 1*. SNQ Duration 1 responses (see Measures) were entered into a matrix labeled “Duration 1.” (d) *Duration of acquaintance 2*. SNQ Duration 2 responses (see Measures) were entered into “Duration 2 giving” and “Duration 2 receiving” matrices.

###### Individual difference variables

We calculated total scores on the FASII and Kiddie Mach, and summed the number of best friends present in the current network. For the IRI, we calculated total scores on the EC and PT subscales. Subsequently, for each year group, we created separate “giving” and “receiving” matrices for each of these five measures by entering scores across matrix rows and columns respectively.

##### Statistical Analysis

The data analysis strategy was as in Study 1. First, statistical tests were conducted to investigate descriptive differences across networks. Second, multiple regressions were conducted to evaluate the impact of network and individual difference variables on mDG investment separately in each network. Third, multigroup multiple regression was conducted to evaluate the impact of network and individual difference variables identified as of potential interest in Steps 1 and 2 on investment across the entire sample, and to pinpoint whether this impact varied as a function of year group, as a proxy for developmental age.

###### Descriptive differences across networks

Analyses of variance (ANOVAs) were conducted comparing mDG money allocated to self, Duration 2, Mach, FASII, IRI PT, and IRI EC across groups, and a chi‐square test was conducted comparing best friends across groups, with statistical significance set at *p *<* *.05 two‐tailed. For comparison of network data (i.e., mDG money allocated to others, SNQ relationship strength, SNQ reciprocation and Duration 1) across groups, ANOVA is not permitted since there is no accepted method for computing whole sample and individual network variance based on nonindependent observations. Therefore, we used the bootstrapping method (see Study 1) to conduct *t* tests comparing network data between all *pairs* of networks (including Year 11A and Year 11B), with Bonferroni correction for multiple comparisons (statistical significance = .05/6, i.e., *p *<* *.0008 two‐tailed).

###### Multiple regression predicting resource allocation within networks

As in Study 1, we used UCINET's MRQAP with double Dekker semipartialing and 10,000 permutations to regress Year 9, Year 10, Year 11A, and Year 11B mDG investment matrices against their respective year group explanatory variables: SNQ relationship strength, SNQ reciprocation, Duration 1, Mach giving, Mach receiving, Duration 2 giving, Duration 2 receiving, FASII giving, FASII receiving, IRI EC giving, IRI EC receiving, IRI PT giving, IRI PT receiving, best friends giving, and best friends receiving. Statistical significance and the strategy for eliminating nonsignificant variables were as in Study 1. Directional hypotheses were as per Study 1 single‐group MRQAP with the addition of the effect on point allocation of the two IRI subscales (Edele et al., 2013).

###### Multiple regression predicting resource allocation across networks

MGMRQAP tested for interactions between school year group (as a proxy for developmental age) and relations between potential explanatory variables and investment in the mDG. As in Study 1, this first entailed the creation of full‐sample matrices for each of the dependent and explanatory variables described above containing data from all participants (*N *=* *81) across the four classes: Years 9, 10, 11A, and 11B. We coded the network level variable “year group” 0, 1, and 2 for participants in Years 9, 10, and 11, respectively. Statistical significance and the strategy for eliminating nonsignificant variables were as in Study 1. Directional hypotheses were as per Study 1 MG‐MRQAP with the addition of the effect on point allocation of IRI subscales. Predictors entered in the initial regression model were (a) variables that differed significantly between groups based on initial ANOVA, chi‐square, or *t*‐test results comparing descriptives between groups; (b) variables that had an impact on investment in any group based on single‐group MRQAP; and (c) terms included to test for interactions with group (for a full list of predictor variables, see content and footnote of Table 6).

### Results

#### Descriptive Differences Across Networks

The four groups did not differ on fictitious money allocated to self, *F*(3, 80) = .96, *p *=* *.415; Mach, *F*(3, 70) = .12, *p *=* *.949; FASII, *F*(3, 78) = 1.57, *p *=* *.203; IRI PT, *F*(3, 78) = .19, *p *=* *.905; or IRI EC, *F*(3, 78) = .04, *p *=* *.987 (see Table 4). Groups differed on the number of years individuals had attended the current school: Duration 2, *F*(3, 79) = 33.82, *p *<* *.001, and the number of self‐reported best friends present in the current network, χ^2^ = 22.64, *p *=* *.007. *T* tests comparing network data between pairs of groups showed differences in money allocated to others (Year 9 vs. all other groups: all three comparisons, *p*s* *<* *.0008), SNQ relationship strength (Year 11A vs. Year 9 and Year 11B: both *p *<* *.0008), SNQ reciprocation (Year 9 vs. Year 10 and Year 11A: both *p*s* *<* *.0008), and Duration 1 (Year 10 vs. Year 11B: *p *<* *.0008; for further details, see Table 4 and Appendix S1). Supplementary Figures S3–S6 depict Years 9, 10, 11A, and 11B networks based on relationship strength.

**Table 4:** Study 2 Descriptives for Each Year Group Network

	Year 9 Midadolescence			Year 10 Later midadolescence			Year 11A Late adolescence			Year 11B Late adolescence			Group comparison
	*M* (*SD*)	Range	*N*	*M* (*SD*)	Range	*N*	*M* (*SD*)	Range	*N*	*M* (*SD*)	Range	*N*	*F*(*df*), *p* or $\chi_{df}^{2}$, *p*
Age (years)	14.10 (0.36)	13.5 to 14.5	13	15.10 (0.38)	14.5 to 16.1	24	15.96 (0.24)	15.5 to 16.4	25	16.00 (0.28)	15.6 to 16.4	19	
Kiddie Mach	56.35 (5.08)	50 to 68	13	57.94 (10.82)	42 to 79	16	57.16 (7.61)	44 to 74	25	56.67 (6.42)	45 to 67	19	*F*(3, 70) = .12, *p *=* *.949
IRI PT	21.61 (3.87)	12 to 26	13	22.37 (5.99)	11 to 31	22	22.80 (4.43)	10 to 29	25	22.82 (5.72)	14 to 35	19	*F*(3, 78) = .19, *p *=* *.905
IRI EC	25.50 (3.82)	18 to 30	13	25.20 (5.56)	11 to 33	22	25.00 (4.82)	14 to 31	25	24.95 (3.75)	16 to 31	19	*F*(3, 78) = .04, *p *=* *.987
FASII	6.54 (2.37)	2 to 9	13	5.87 (2.01)	0 to 9	22	6.80 (1.56)	4 to 9	25	7.00 (1.50)	4 to 9	18	*F*(3, 78) = 1.57, *p *=* *.203
Duration 2	2.50 (0.00)	2.50 to 2.50	13	3.25 (0.74)	0.5 to 3.5	24	4.46 (0.20)	3.5 to 4.5	25	4.34 (0.69)	1.5 to 4.5	19	*F*(3, 80) = 53.20, *p *<* *.001
Best friends	0.91 (0.70)	0 to 2	12	0.96 (0.93)	0 to 3	23	0.80 (0.96)	0 to 3	25	1.00 (1.05)	0 to 3	19	χ^2^(9) = 22.64, *p *=* *.007
mDG: Points to self	20.29 (18.98)	0 to 58.82	13	33.08 (32.33)	0 to 100	24	36.79 (30.24)	0 to 90	25	31.29 (27.53)	0 to 100	19	*F*(3, 80) = .96, *p *=* *.415

	Density M (SEM)	Range	*N*	Density M (SEM)	Range	*N*	Density M (SEM)	Range	*N*	Density M (SEM)	Range	*N*	*t*‐test group difference a
mDG: Points to others	6.64 (.42)	0 to 30	156	2.78 (.24)	0 to 50	552	2.63 (.28)	0 to 60	600	3.82 (.46)	0 to 100	342	Year 9 versus all other groups
Relationship strength	0.22 (.03)	−.25 to 1	156	0.15 (.01)	−.33 to 1	552	0.10 (.01)	−.33 to 1	600	0.24 (.02)	−.33 to 1	342	Year 9 versus Year 11A and Year 11B
Relationship reciprocation	0.21 (.01)	−.81 to .81	156	0.15 (.01)	−.67 to .67	552	0.14 (.01)	−.78 to .78	600	0.16 (.01)	−.63 to .63	342	Year 9 versus Year 10 and Year 11A
Duration 1	0.12 (.03)	0 to 1	156	0.15 (.02)	0 to 1	552	0.10 (.01)	0 to 1	600	0.06 (.01)	0 to 1	342	Year 10 versus Year 11B

Note

The upper half of the table shows nonnetwork descriptives while the lower half of the table shows descriptives for network data. Network data were compared across groups using *t* tests based on bootstrapped mean density and standard error, with degrees of freedom based on *N* observations across groups. IRI PT/EC = interpersonal reactivity index PT/EC subscales; FASII = Family Affluence Scale; Duration 2 = for how many years the participant had attended the current school; Best friends = number of self‐nominated best friends in the current network; mDG = modified Dictator Game; Duration 1 = whether the participant had known his or her classmate since primary (= 1) or secondary (= 0) school.

^a^For comparison of network data across groups, analyses of variance is not permitted since there is no accepted method for computing whole sample and individual network variance based on nonindependent observations. Therefore, we used the bootstrapping method (see Methods in Study 1) to conduct *t* tests comparing network data between all pairs of networks, with Bonferroni correction for multiple comparisons (statistical significance = .05/6, i.e., *p *<* *.0008 two‐tailed). For *t* and *p* values, see Appendix S1.

#### Multiple Regression Predicting Resource Allocation Within Networks

We regressed each year group's mDG allocation matrix on their explanatory variable matrices using MRQAP yielding final models shown in Table 5. For Year 9, *R* ^2^_adj_ = .47, *p *<* *.001; for Year 10, *R* ^2^_adj_ = .39, *p *<* *.001; for Year 11A, *R* ^2^_adj_ = .39, *p *<* *.001; and for Year 11B, *R* ^2^_adj_ = .39, *p *<* *.001. SNQ relationship strength was significant in all networks (Year 9: *b *=* *10.37, *p *<* *.001; Year 10: *b *=* *12.16, *p *<* *.001; Year 11A: *b *=* *15.87, *p *<* *.001; Year 11B: *b *=* *19.84, *p *<* *.001). SNQ reciprocation was significant in Year 10, Year 11A, and Year 11B networks only (Year 10: *b *=* *3.03, *p *=* *.002; Year 11A: *b *=* *3.72, *p *=* *.002; Year 11B: *b *=* *6.52, *p *<* *.001). The impact of IRI EC on point allocation was significant in Year 9 only (*b *=* *.23, *p *=* *.023), and the impact of Duration 1 was significant in Year 10 only (*b *=* *−1.64, *p *=* *.010). Ceteris paribus, participants in all networks gave more points to those towards whom they reported stronger social ties. In Year 10 and both Year 11 networks, ceteris paribus, participants gave more to individuals who positively reciprocated social ties. In Year 9, ceteris paribus, participants who scored higher on the IRI EC subscale gave more to their classmates, and in Year 10, participants gave less to individuals they had known since primary versus secondary school (see Table 5).

**Table 5:** Study 2 Multiple Regression Models Predicting Resource Allocation Within Networks

Independent variable	Unstandardized regression coefficient	Standardized regression coefficient	*p* value
Year 9: Midadolescence			
MRQAP final model: *R* ^2^_adj_ = .47, *p *< .001			
Intercept	0.74	.00	< .001
Relationship strength	10.37	.66	< .001^a^
Empathic concern giving	0.23	.16	.023^a^
Year 10: Later midadolescence			
MRQAP final model: *R* ^2^_adj_ = .39, *p *<* *.001			
Intercept	1.23	.00	< .001
Relationship strength	12.16	.67	< .001^a^
Reciprocation	3.03	.11	.002^a^
Duration 1	−1.64	−.11	.010
Year 11A: Late adolescence			
MRQAP final model: *R* ^2^_adj_ = .39, *p *< .001			
Intercept	1.14	.00	< .001
Relationship strength	15.87	.66	< .001^a^
Reciprocation	3.72	.11	.002^a^
Year 11B: Late adolescence			
MRQAP final model: *R* ^2^_adj_ = .39, *p *<* *.001			
Intercept	−0.72	.00	< .001
Relationship strength	19.84	.68	< .001^a^
Reciprocation	6.52	.16	< .001^a^

Note

Multiple regression with quadratic assignment procedure (MRQAP) was used to predict modified Dictator Game point allocation based on individual difference and network variables. Independent variables eliminated from the Year 9 model due to nonsignificance were IRI EC receiving (*b *=* *.01, *p *=* *.465), IRI PT receiving (*b *=* *.01, *p *=* *.471), best friends receiving (*b *=* *.07, *p *=* *.438), Mach giving (*b *= −.02, *p *=* *.429), best friends giving (*b *= −.27, *p *=* *.339), Duration 1 (*b *= −.40, *p *=* *.336), IRI PT giving (*b *= −.06, *p *=* *.275), FASII receiving (*b *= −.08, *p *=* *.262), SNQ reciprocation (*b *=* *.90, *p *=* *.252), Mach receiving (*b *= −.09, *p *=* *.082), and FASII giving (*b *= −.33, *p *=* *.054). Due to low variance, Duration 2 variables were not entered into the model. Independent variables eliminated from the Year 10 model were Mach giving (*b *=* *.00, *p *=* *.485), IRI EC receiving (*b *=* *.00, *p *=* *.468), best friends giving (*b *=* *.05, *p *=* *.470), FASII giving (*b *=* *.02, *p *=* *.445), IRI PT giving (*b *= −.09, *p *=* *.131), IRI EC giving (*b *=* *.04, *p *=* *.207), Duration 2 receiving (*b *=* *.29, *p *=* *.121), best friends receiving (*b *= −.19, *p *=* *.150), Duration 2 giving (*b *= −.65, *p *=* *.091), IRI PT receiving (*b *=* *.02, *p *=* *.074), FASII receiving (*b *= −.12, *p *=* *.059), and Mach receiving (*b *=* *.01, *p *=* *.091). Independent variables eliminated from the Year 11A model were IRI EC receiving (*b *= −.01, *p *=* *.446), IRI EC giving (*b *= −.02, *p *=* *.437), FASII giving (*b *=* *.06, *p *=* *.403), best friends giving (*b *=* *.06, *p *=* *.424), FASII receiving (*b *= −.10, *p *=* *.307), best friends receiving (*b *=* *−.13, *p *=* *.313), IRI PT giving (*b *=* *−.08, *p *=* *.183), Mach giving (*b *=* *−.02, *p *=* *.328), IRI PT receiving (*b *=* *−.11, *p *=* *.063), Mach receiving (*b *=* *−.03, *p *=* *.182), and Duration 1 (*b *=* *−1.26, *p *=* *.072). Due to low variance, Duration 2 variables were not entered into the model. Independent variables eliminated from the Year 11B model were FASII receiving (*b *=* *−.02, *p *=* *.461), IRI EC giving (*b *=* *.02, *p *=* *.457), Mach giving (*b *=* *.01, *p *=* *.405), FASII giving (*b *=* *−.06, *p *=* *.382), IRI PT giving (*b *=* *−.04, *p *=* *.310), Mach receiving (*b *=* *−.01, *p *=* *.317), IRI EC receiving (*b *=* *.05, *p *=* *.276), best friends receiving (*b *=* *−.18, *p *=* *.261), best friends giving (*b *=* *−.39, *p *=* *.156), IRI PT receiving (*b *=* *−.07, *p *=* *.076), and Duration 1 (*b *=* *−2.18, *p *=* *.090). Due to low variance, Duration 2 variables were not entered into the model. SNQ = Social Network Questionnaire; IRI PT = interpersonal reactivity index perspective‐taking; EC = empathic concern; FASII = family Affluence Scale.

^a^Denotes a one‐tailed *p* value; all other *p* values are two‐tailed.

#### Multiple Regression Predicting Resource Allocation Across Networks

We regressed mDG allocation across the entire sample (*N *=* *81) on explanatory variable matrices using MG‐MRQAP. In the final point allocation model (*R* ^2^_adj_ = .43, *p *<* *.001), significant predictor variables were SNQ relationship strength (*b *=* *9.45, *p *<* *.001), SNQ Relationship Strength × Year Group (*b *=* *3.88, *p *<* *.001), SNQ Reciprocation × Year Group (*b *=* *2.15, *p *=* *.010), Duration 1 (*b *=* *−1.32, *p *=* *.014), IRI EC giving (*b *=* *.22, *p *=* *.006), and IRI EC Giving × Year Group (*b *= −.13, *p *=* *.026; see Table 6). Ceteris paribus, participants gave more fictitious money to those to whom they reported stronger social ties, with this effect being greater in older year groups. Relative to younger year groups, ceteris paribus, participants in older year groups gave more to individuals who positively reciprocated social ties. All else being equal, participants who scored higher on IRI EC gave more, with this effect greater in younger networks, while participants gave less to individuals they had known since primary versus secondary school.

**Table 6:** Study 2 Multiple Regression Models Predicting Resource Allocation Across Networks

Independent variable	Unstandardized regression coefficient	*p* value
MG‐MRQAP model		
*R* ^2^_adj_ = .43, *p *<* *.001		
Year 9 intercept	0.53	.006
Year 10 intercept (reference Year 9)	−1.52	.126
Year 11A intercept (reference Year 9)	−1.88	.480
Year 11B intercept (reference Year 9)	1.01	< .001
Relationship strength	9.45	< .001^a^
Relationship Strength × Year Group	3.88	< .001^a^
Reciprocation	0.62	.658
Reciprocation × Year Group	2.15	.010^a^
Duration 1	−1.32	.014
Empathic concern giving	0.22	.006^a^
Empathic Concern Giving × Year Group	−0.13	.026

Note

Multigroup multiple regression with quadratic assignment procedure (MG‐MRQAP) was implemented to predict modified Dictator Game point allocation based on individual difference and network variables and terms specifying their respective interactions with year group (Year 9, Year 10, and Year 11). Independent variables eliminated due to nonsignificance were Best Friends Giving × Group (*b *=* *.04, *p *=* *.552), best friends giving (*b *=* *.00, *p *=* *.505), Best Friends Receiving × Group (*b *=* *.05, *p *=* *.554), Duration 1 × Group (*b *= −.26, *p *=* *.396), Duration 2 Giving × Group (*b *=* *.26, *p *=* *.661), best friends receiving (*b *= −.08, *p *=* *.316), Duration 2 Receiving × Group (*b *= −.61, *p *=* *.112), Duration 2 receiving (*b *= −.03, *p *=* *.440), and Duration 2 giving (*b *= −.46, *p *=* *.071). Note that the nonsignificant variable reciprocation is retained to examine its interaction with year group.

^a^Denotes a one‐tailed *p* value; all other *p* values are two‐tailed.

### Discussion

Study 2 was conducted to replicate findings from Study 1, and to extend them by investigating hypothesized development between mid‐ to late adolescence in the impact of reciprocation on mDG resource allocation. Thus, Study 2 compared three developmental time points to evaluate evidence for an effect of school year group, as a proxy for developmental age, on the relation between the social network variable reciprocation and mDG investment. Replicating Study 1 results, we found first that relationship strength modulated investment in all networks, whereas reciprocation modulated investment only in older networks (Years 10 and 11). Second, we found that with increasing age group, reciprocation exerted an *increasing* impact on investment. It is notable that such findings were obtained while taking into account potentially confounding variables such as duration of acquaintance, the number of best friends present in the current network, and familial levels of wealth.

In Study 2, we observed an interaction between year group and SNQ relationship strength that was not present in Study 1. That is, with increasing year group, resource allocation was increasingly modulated by the social tie strength reported by a participant toward his or her classmates, such that participants in older year groups were increasingly likely to give more to those to whom they reported a stronger social tie. Further studies are needed to replicate and confirm this novel age group difference in the impact of social tie strength on investment.

Some descriptive differences were observed across groups in Study 2. Duration 2, that is, for how many years the participant had attended the current school, was greater in older year groups (in contrast to greater mean duration of acquaintance in the younger vs. older group in Study 1). Additionally, the number of self‐reported best friends present in the current network differed significantly across groups. However, since these variables were eliminated from the full sample regression model, they cannot be said to account for age group differences in patterns of investment. Relationship strength and reciprocation differed across groups (see Table 4 and Appendix S1), but not in a manner that was systematically related to age group. Fictitious money invested in others differed across groups; however, this difference is taken into account statistically within the multigroup model, since in a fixed effects approach by groups the intercept (amount invested in other network members if all independent variables are equal to 0) is allowed to vary between groups.

In addition to the above‐mentioned effects of age group and the SNQ variables relationship strength and reciprocation on resource allocation, single‐ and multigroup models revealed effects of a number of additional variables. In the youngest network (midadolescent: Year 9), participants who scored higher on a measure of self‐reported empathy (IRI EC) invested more in peers relative to those who scored lower on this measure (ceteris paribus), whereas this variable had no impact in older year groups (Table 5). The significant interaction between IRI EC and age group on investment in the multigroup model (Table 6) can be interpreted as indicating that the impact of this variable decreased with increasing year group. Although this result requires replication, younger participants were potentially utilizing an investment strategy based on their own feelings of empathy rather than on an appraisal of both self and other's views on each dyadic relationship. This interpretation is consistent with Selman's (1980) account of the development of perspective taking, in that midadolescents (aged 12–14 years) commonly engage in simple reciprocal perspective taking that takes into account self *or* other's perspective (potentially, one's own feelings of empathy), whereas older adolescents are more likely to simultaneously take into account self *and* other's perspectives on the relationship.

Another variable that exerted effects on giving in single and multigroup models was Duration 1 (whether participants had been mutually acquainted since primary vs. secondary school). Ceteris paribus, participants in the Year 10 network invested *less* in individuals to whom they had been acquainted since primary versus secondary school, and this effect was also evident in the multigroup model. That is, across the sample, participants gave less to peers to whom they had been acquainted for longer. However, since an analogous variable (duration of acquaintance in years) had no impact on investment in Study 1, this result requires replication before further conclusions can be drawn. Finally, Study 2 failed to find an effect of Machiavellian orientation (Kiddie Mach) on investment as observed in Study 1, either in single‐group or multigroup models, which suggests that this finding may not be generalizable.

## General Discussion

The current set of studies investigated development during adolescence in the impact of relationship reciprocation on resource allocation within established peer networks. For the first time, we show evidence for continuing development between mid‐ and late adolescence in the extent to which reciprocation of authentic social ties predicts prosocial cooperation behavior.

Across both studies, individual network regression (MRQAP) models were consistent with hypothesized age group differences in the impact of reciprocation. In midadolescence (*M*_age_ = 14), individuals invested more in peers to whom they reported stronger social ties (i.e., trust, liking, feelings of companionship). In later adolescence (ages = 15–17), individuals additionally invested more in peers who *reciprocated* strong ties. That is, late adolescents, but not midadolescents, were behaviorally sensitive to the degree to which others reciprocated their feelings of friendship, and adjusted their investment strategy accordingly.

In both studies, results from full‐sample regression (MG‐MRQAP) models showed that with increasing grade level, participants showed an increasing tendency to give more to classmates who reported a stronger relationship toward themselves than they reported toward their classmates. That is, between mid‐ and late adolescence (ages = 14–17), individuals' resource allocation decisions showed *increasing* sensitivity to the degree of relationship reciprocation. It is noteworthy that these convergent findings were obtained despite differences across studies in participant characteristics (private vs. state school; mixed‐ vs. single‐gender networks), and differences in the mDG (e.g., use of real vs. fictitious money).

The current findings are consistent with prior developmental evidence from social‐cognition and game theory paradigms suggesting that the tendency to take into account an interactant's perspective, motivations, and contributions in an experimental context continues to develop until at least midadolescence (Almås et al., 2010; Dumontheil et al., 2010; Eisenberg, Carlo, Murphy, & Van Court, 1995; Güroğlu et al., 2009; Van den Bos et al., 2010; Van Lange, Otten, De Bruin, & Joireman, 1997). In the current study, late adolescents, but not midadolescents, were able to use information regarding relationship reciprocation in resource allocation decisions. In contrast, midadolescent participants' resource allocation was determined by social tie strength (their own feelings of friendship) and (in Study 2) self‐reported empathy. Potentially, older adolescents, with their more advanced perspective‐taking abilities, were able to use information regarding their classmates' perceptions of the dyadic relationships to guide their investment decisions. In contrast, investment decisions of younger participants showed no evidence of sensitivity to the other's perspective on the relationship. These results are therefore also broadly consistent with the suggestion that social cognitive perspective taking shows protracted development during adolescence, particularly as assessed in the context of relationships with peers (Gurucharri & Selman, 1982; Selman, 1980). An important question is whether midadolescents were able to perceive relationship reciprocation subjectively but failed to use this information behaviorally (cf. Keysar, Lin, & Barr, 2003). Future studies are needed to unravel the direction of causality and to provide a broader framework within which to interpret the data. Studies should also investigate potential relations between the current findings and qualitative and quantitative observations of adolescent peer relationships (Bigelow & La Gaipa, 1980; Urberg et al., 1995; Youniss, 1982).

A number of additional points may be of note. Although the current studies investigated relationships within a number of school classes, in reality participants also have many relationships outside these classes. A common technique for investigating social networks based on peer nominations accounts for this complexity by asking each participant to name, for example, two close friends, and then asking these purported close friends to do the same, and so on (Marsden, 1990). However, although this technique has yielded important insights, it does not systematically map dyadic relationships bidirectionally and as such may be less interesting with regard to underlying social cognitive variables such as perspective taking. In contrast, because the method used in the current study maps dyadic ties exhaustively in a closed network (i.e., it maps each participant's relationship with each other participant), it provides information about bidirectional ties and thus reciprocation.

The current findings raise some interesting possibilities. First, maturation of behavioral sensitivity to reciprocity could contribute to important changes in peer relationships observed across adolescence. For example, a more nuanced understanding of bidirectional relationship qualities could contribute to developmental changes in expectations of friendships, and thus underpin observed decreases across adolescence in the number of self‐reported close friendships (Urberg et al., 1995) and the increase in emphasis on intimacy and reciprocity (Youniss, 1982). Further studies utilizing the combination of experimental social‐cognition tasks and mapping of real‐world peer relationships may shed further light on this hypothesis. Second, the approach adopted here, which relates social network variables to an experimental measure of social behavior (Harrison et al., 2011), could potentially be utilized in neuroimaging research on dynamic social interactions and enduring social relationships, revealing general principles relating social cognitive and social network variables to their biological basis in the brain (Kanai, Bahrami, Roylance, & Rees, 2011; Lewis, Rezaie, Brown, Roberts, & Dunbar, 2011; Schilbach et al., 2013).

Finally, the current study illuminates a potentially important aspect of building positive social relationships, which are an important contributor to long‐term mental health (House, Landis, & Umberson, 1988). Learning to invest in individuals who reciprocate prosocial behavior may promote more sustainable relationships, which in turn serve as a protective factor against mental illness. The ability to reciprocate cooperative behavior enables individuals to integrate as functioning members of social groups, while those who fail to do so may be punished and alienated (El Mouden, West, & Gardner, 2010; Fehr & Gächter, 2002). Understanding these sociocognitive processes during adolescence, which is a period of vulnerability to adverse mental health outcomes, could potentially be of value (Clifton, Pilkonis, & McCarty, 2007; Ormel et al., 2014; Paus, Keshavan, & Giedd, 2008).

### Conclusions

The present set of studies used quantitative social network techniques to examine bidirectional relationship characteristics relevant for understanding subtle changes in cooperative behavior across adolescence. Results suggest development between mid‐ and late adolescence in the behavioral ability to recognize the degree to which others reciprocate feelings of friendship, in terms of impact on resource allocation in an mDG. These data underscore the importance of taking into account features of authentic relationships in research on adolescent prosocial behavior. Broadly, the mastery of sophisticated social strategies within adolescence may be critical for navigating an increasingly complex social world (Brown & Larson, 2009; Nelson et al., 2005). Combining social network methodology with experimental indices of prosocial behavior (Harrison et al., 2011) may reveal further insights into the development of strategic prosociality, its basis in the maturing adolescent brain, and its contribution to resilience across the life span.

## Supporting information


**Figure S1.** Study 1, Year 9 (Mid‐Adolescent) Network. *N* = 23, *M* (*SD*) age = 14.46 (.26).
**Figure S2.** Study 1, Year 12 (late adolescent) Network. *N* = 19, *M* (*SD*) age = 17.22 (.29).
**Figure S3.** Study 2, Year 9 (Mid‐Adolescent) Network. *N* = 13, *M* (*SD*) age = 14.10 (.36).
**Figure S4.** Study 2, Year 10 (Later Mid‐Adolescent) Network. *N* = 24, *M* (*SD*) Age = 15.10 (.38).
**Figure S5.** Study 2, Year 11A (Late Adolescent) Network. *N* = 25, *M* (*SD*) Age = 15.96 (.24).
**Figure S6.** Study 2, Year 11B (Late Adolescent) Network. *N* = 19, *M* (*SD*) age = 16.00 (.28).
**Appendix S1.** Study 2 Network Data: *t*‐Tests Comparing Pairs of Groups.
