# Supplementary material for: Relationship Reciprocation Modulates Resource Allocation in Adolescent Social Networks: Developmental Effects
Source: Child Dev. 2015 Jul 31;86(5):1489–506. doi: 10.1111/cdev.12396 (PMC4855684; doi:10.1111/cdev.12396)

**Supporting Info for Online Publication Only**

**Appendix S1: Study 2 network data: t-tests comparing pairs of groups.**

For comparison of network data across groups, ANOVA is not permitted since there is no accepted method for computing whole sample and individual network variance based on non-independent observations. We used the bootstrapping method (see **Study 1** Methods) to conduct *t*-tests comparing the following network data between all pairs of groups, with Bonferroni correction (statistical significance = 0.05/6 i.e. *p*<.0008):

mDG points to others:

Year 9 vs. 10: *t*_706_=7.97, *p*<.0008

Year 9 vs. 11A: *t*_754_=7.92, *p*<.0008

Year 9 vs. 11B: *t*_496_=4.51, *p*<.0008

Year 10 vs. 11A: *t*_1150_=.412, *p*=.340

Year 10 vs. 11B: *t*_892_=2.01, *p*=.420

Year 11A vs. 11B: *t*_940_=1.90, *p*=.029

SNQ relationship strength:

Year 9 vs. 10: *t*_706_=2.31, *p*=.189

Year 9 vs. 11A: *t*_754_=3.94, *p*<.0008

Year 9 vs. 11B: *t*_496_=.528, *p*=.299

Year 10 vs. 11A: *t*_1150_=2.67, *p*=.003

Year 10 vs. 11B: *t*_892_=4.19, *p*=.004

Year 11A vs. 11B: *t*_940_=4.22, *p*<.0008

SNQ relationship reciprocation:

Year 9 vs. 10: *t*_706_=3.88, *p*<.0008

Year 9 vs. 11A: *t*_754_=4.59, *p*<.0008

Year 9 vs. 11B: *t*_496_=3.11, *p*=.0010

Year 10 vs. 11A: *t*_1150_=1.41, *p*=.079

Year 10 vs. 11B: *t*_892_=1.21, *p*=.113

Year 11A vs. 11B: *t*_940_=1.38, *p*=.084

Duration 1:

Year 9 vs. 10: *t*_706_=.88, *p*=.189

Year 9 vs. 11A: *t*_754_=.64, *p*=.262

Year 9 vs. 11B: *t*_496_=2.06, *p*=.020

Year 10 vs. 11A: *t*_1150_=2.31, *p*=.011

Year 10 vs. 11B: *t*_892_=4.36, *p*<.008

Year 11A vs. 11B: *t*_940_=1.43, *p*=.077

**Supplementary Figurs.** All figures are illustrative. Relationship strength is represented by edge thickness: Stronger social ties are represented by thicker edges. Circles indicate females, squares indicate males. Node distance is based on NetDraw iterative metric multidimensional scaling in UCINET (Borgatti et al., 2002) and represents similarity of social ties.

**Figure S1.** Study 1, Year 9 (mid-adolescent) network. N=23, mean (SD) age=14.46 (.26).

**
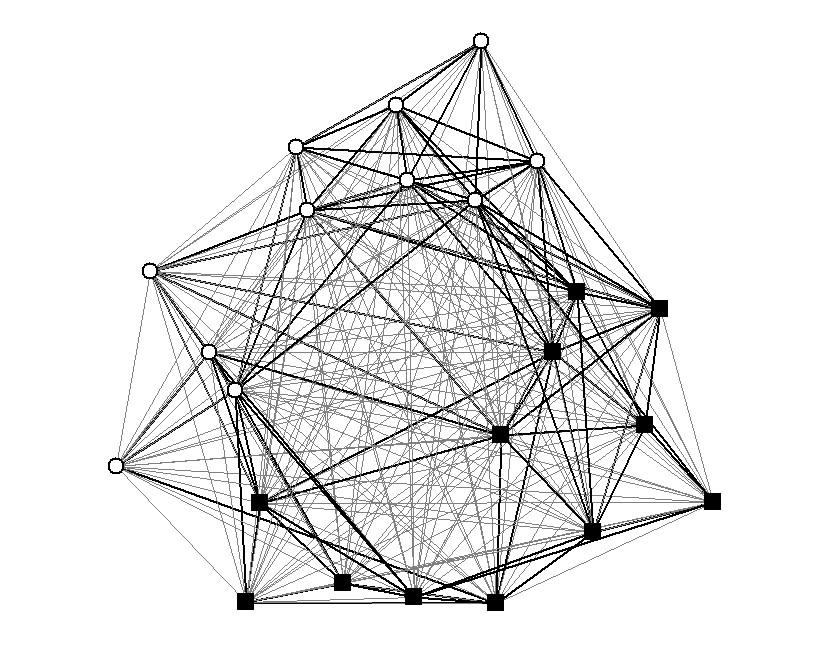
**

**Figure S2.** Study 1, Year 12 (late adolescent) network. N=19, mean (SD) age=17.22 (.29).


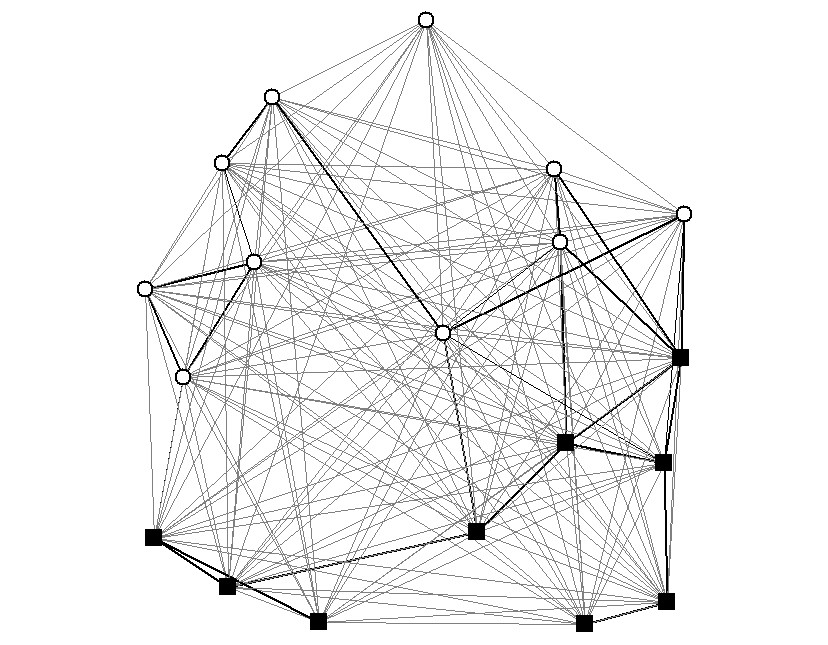


**Figure S3.** Study 2, Year 9 (mid-adolescent) network. N=13, mean (SD) age=14.10 (.36).

**
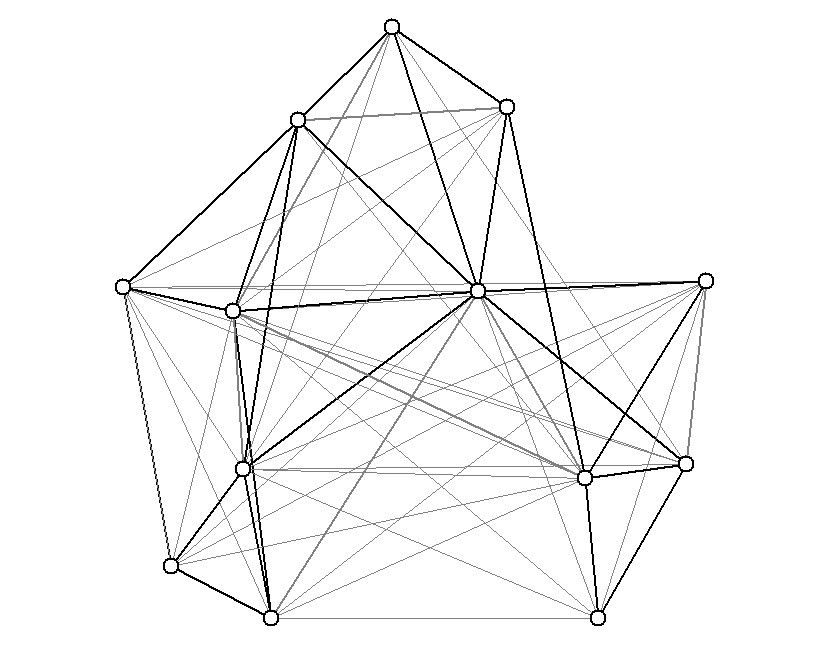
**

**Figure S4.** Study 2, Year 10 (later mid-adolescent) network. N=24, mean (SD) age=15.10 (.38).

**
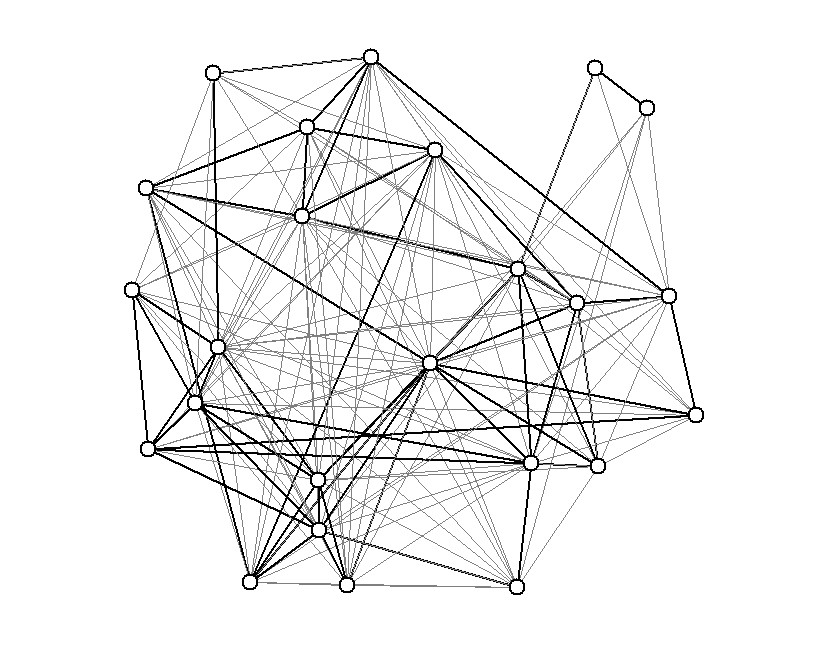
**

**Figure S5.** Study 2, Year 11A (late adolescent) network. N=25, mean (SD) age=15.96 (.24).

**
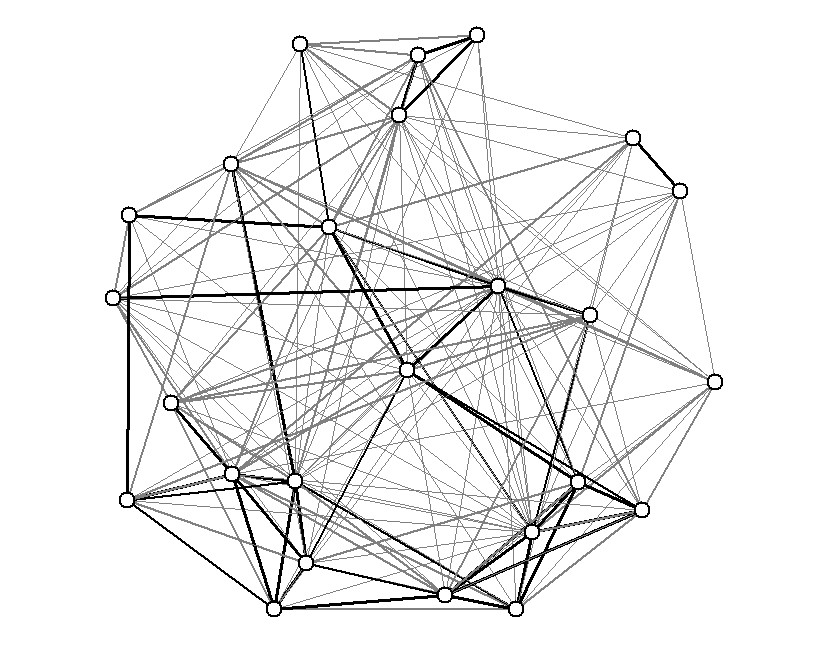
**

**Figure S6.** Study 2, Year 11B (late adolescent) network. N=19, mean (SD) age=16.00 (.28).


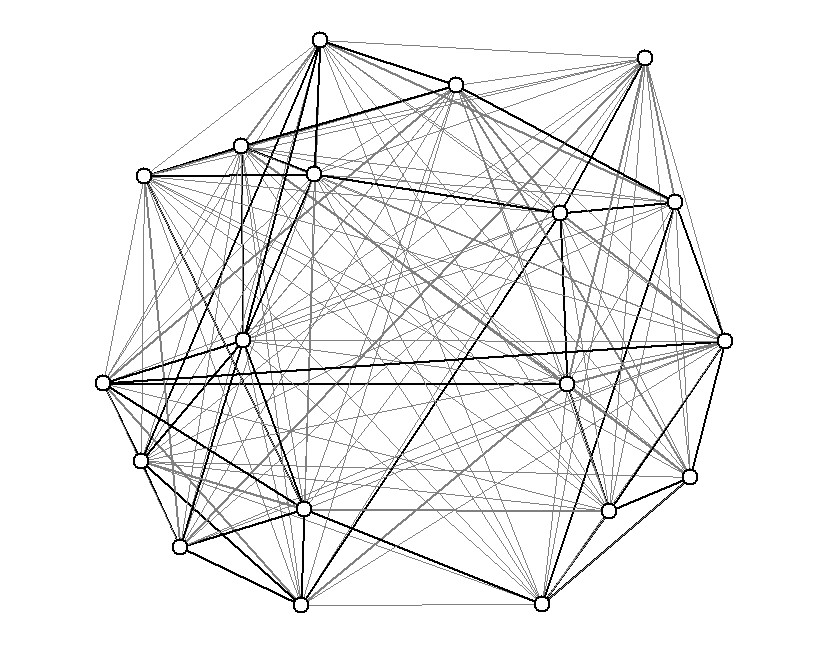

Supplement: Supplementary file 1 — Figure S1. Study 1, Year 9 (Mid‐Adolescent) Network. N = 23, M (SD) age = 14.46 (.26). Figure S2. Study 1, Year 12 (late adolescent) Network. N = 19, M (SD) age = 17.22 (.29). Figure S3. Study 2, Year 9 (Mid‐Adolescent) Network. N = 13, M (SD) age = 14.10 (.36). Figure S4. Study 2, Year 10 (Later Mid‐Adolescent) Network. N = 24, M (SD) Age = 15.10 (.38). Figure S5. Study 2, Year 11A (Late Adolescent) Network. N = 25, M (SD) Age = 15.96 (.24). Figure S6. Study 2, Year 11B (Late Adolescent) Network. N = 19, M (SD) age = 16.00 (.28). Appendix S1. Study 2 Network Data: t‐Tests Comparing Pairs of Groups. [file CDEV-86-1489-s001.docx]
